# TBC2target: A Resource of Predicted Target Genes of Tea Bioactive Compounds

**DOI:** 10.3389/fpls.2018.00211

**Published:** 2018-02-22

**Authors:** Shihua Zhang, Liang Zhang, Yijun Wang, Jian Yang, Mingzhi Liao, Shoudong Bi, Zhongwen Xie, Chi-Tang Ho, Xiaochun Wan

**Affiliations:** ^1^State Key Laboratory of Tea Plant Biology and Utilization, Institute of Applied Mathematics, Anhui Agricultural University, Hefei, China; ^2^Department of Genetics, University of Georgia, Athens, GA, United States; ^3^College of Life Sciences, Northwest A&F University, Xianyang, China; ^4^Department of Food Science, Rutgers University, New Brunswick, NJ, United States

**Keywords:** tea bioactive compound, target gene, healthy benefit of tea, network, database

## Abstract

Tea is one of the most popular non-alcoholic beverages consumed worldwide. Numerous bioactive constituents of tea were confirmed to possess healthy benefits via the mechanisms of regulating gene expressions or protein activities. However, a complete interacting profile between tea bioactive compounds (TBCs) and their target genes is lacking, which put an obstacle in the study of healthy function of tea. To fill this gap, we developed a database of target genes of TBCs (TBC2target, http://camellia.ahau.edu.cn/TBC2target) based on a pharmacophore mapping approach. In TBC2target, 6,226 interactions between 240 TBCs and 673 target genes were documented. TBC2target contains detailed information about each interacting entry, such as TBC, CAS number, PubChem CID, source of compound (e.g., green, black), compound type, target gene(s) of TBC, gene symbol, gene ID, ENSEMBL ID, PDB ID, TBC bioactivity and the reference. Using the TBC-target associations, we constructed a bipartite network and provided users the global network and local sub-network visualization and topological analyses. The entire database is free for online browsing, searching and downloading. In addition, TBC2target provides a BLAST search function to facilitate use of the database. The particular strengths of TBC2target are the inclusion of the comprehensive TBC-target interactions, and the capacity to visualize and analyze the interacting networks, which may help uncovering the beneficial effects of tea on human health as a central resource in tea health community.

## Introduction

Tea, produced from the dried leaves of tea plant (*Camellia sinensis*) is one of the most popular non-alcoholic beverages consumed worldwide (Tai et al., 2015). Considerable studies have confirmed the critical health effects (e.g., anti-inflammation, cancer prevention) of tea due to its ample bioactive small-molecular components, such as flavan-3-ols, flavanonol, phenolic acids, alkaloids, proanthocyanidins, fatty acids, terpenoids, carbohydrates, and amino acids (Zhou et al., 2012a,b; Huang et al., 2013; Zhang et al., 2013, 2015; Weerawatanakorn et al., 2015; Han et al., 2016; Yang et al., 2016). In the past decades, many tea bioactive compounds (TBCs) have been found to possess multifarious beneficial effects through regulating gene expressions or protein activities, such as the anti-inflammatory potential of (-)-epigallocatechin gallate (EGCG) through the inhibition of TNF-α and NF-kB expression in mouse macrophage cell line (Yang et al., 1998), the anti-tumor effects of theaflavin-3′-gallate through the activation of GST, GPx, SOD, and CAT in murine skin carcinogenesis model (Saha and Das, 2002), the hepatoprotective effect of quercetin-3-*O*-glucosylrhamnosylgalactoside through the inhibition of Gpt and Got2 expression in liver injuried rats (Wada et al., 2000), and the anti-obesity effect of caffeine through the inhibition of SIRT1, CEBP, and CEBPD expression in human preadipocytes and adipocytes (Sohle et al., 2009).

Despite many significant results have been achieved in dissecting TBC and target gene interactions related to tea beneficial effects on health, the health-promoting mechanisms of tea is still not fully understood. It is noted that previous studies mostly used low-throughput technologies, such as quantitative PCR and Northern Blot, to identify target gene(s) of certain TBCs (Klein and Fischer, 2002; Chen C.N. et al., 2005; Chen D. et al., 2005; Li et al., 2016). Therefore, a complete interacting profile between TBCs and their target genes is lacking, which limits the scope of their application in dissection of tea healthy function. In addition, published experimental data has shown that different TBCs may synergistically target the same/different target gene(s) and trigger the similar/different health-promoting effects (Lee and Yen, 2006; Miura et al., 2001). In a recent effort, Zhang et al. (2014) *in silico* analyzed the compound-target-disease network of fifteen green tea polyphenolics (GTPs) and disclosed that GTPs act on different target genes in the signaling network of complex disease via a synergistic fashion. These findings are evidently in accordance with the holistic vision of network pharmacology that follows the “multicomponent, network target” model (Hopkins, 2007). On the whole, it is promising to systematically analyze the healthy mechanisms of tea based on a predicted TBC-target association data using the above mentioned network pharmacology approach.

With those considerations, we developed a database named “TBC2target” for target genes of TBCs using a pharmacophore mapping based prediction. TBC2target archived 6,226 predicted interactions between 240 TBCs and 673 target genes. Detailed information about each TBC-target interacting entry such as TBC, CAS number, PubChem CID, source of compound (e.g., green, black), compound type, target gene(s) of TBC, gene symbol, gene ID, ENSEMBL ID, PDB ID, TBC bioactivity and the reference were provided. Despite of the fundamental browse, search, and download functions, we also deployed several useful applications, such as network visualization and topological analysis and BLAST search, in this database for users. Therefore, we believe TBC2target will serve as a valuable and central resource for the study of healthy mechanisms of tea.

## Materials and Methods

### TBCs Collecting and 3D Structure Drawing

We used the curation pipeline, described in our previously published TBC2health project (Zhang et al., 2016), to collect experimentally validated TBC entries. It is notable that TBC2health-recorded TBCs originated from both tea infusions and plant parts. In TBC2target, we focused on the interactions between tea infusions and human health. Therefore, TBC entries from different tea infusions were considered in this study. Based on this scheme, a total of 240 TBCs were collected. For these TBCs, a chemist manually produced their 3D structures using ISIS Draw^1^ (MDL Information Systems, Inc.) by referring to the original articles. The 3D structures of TBCs were optimized in Sybyl^2^ (Tripos, Inc.) using the standard Tripos force field (Zhang et al., 2011).

### Target Genes Prediction of TBCs

With 3D structures of TBCs available, we used the web server PharmMapper to predict target genes of TBCs. PharmMapper (Liu et al., 2010) is designed based on a pharmacophore mapping strategy to accurately identify potential target genes using a small molecule as query in its background database, which is a large-scale pharmacophore repertoire curated from target information in TargetBank (in-house data in PharmMapper project), DrugBank (Wishart et al., 2008), BindingDB (Liu et al., 2007), and PDTD (Gao et al., 2008). The server PharmMapper can help to find the optimal mapping poses of the user-uploaded small molecules against all the target genes in PharmTargetDB and the top *N* potential candidates together with the respective molecules’ aligned poses are outputted. In this study, the targets with a Fit Score value higher than 4.000 were selected as potential targets to ensure the high-confidence of TBC-target interactions (Chen, 2014).

### Network Visualization and Analysis

**FIGURE 1:**
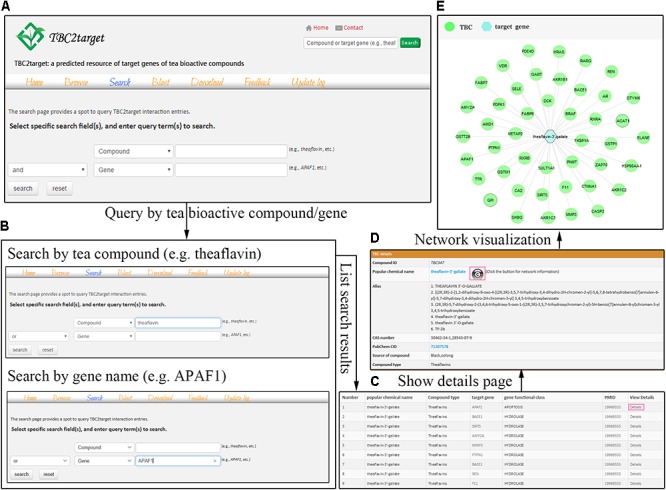
The search interface of TBC2target. In the search page, users can conduct logical and fuzzy search using compound and gene fields separately or cooperatively **(A)**. Two examples that used theaflavin and APAF1 were shown in this page **(B)**. For specific theaflavin-3′-gallate, the direct interactions were visualized in a network fashion by a button clicking in the details page **(C–E)**.

A bipartite network was constructed to describe the predicted TBC-target interactions. In this network, a node represented a TBC or a gene, and an edge represented the interacting relationship between a TBC and a gene. We used the Cytoscape Web (Lopes et al., 2010) to present a network visualization interface, from which the global TBC-target network and direct interacting sub-network of a TBC or a gene can be displayed for users. In the network visualization, TBC and gene were marked with different shapes and colors (**Figure 1E**). To further topological analysis of the network, several typical parameters, such as degree, betweenness, radiality and neighborhood, were computed for a TBC or a gene using the Cytoscape plugin NetworkAnalyzer (Assenov et al., 2008).

### Information Annotation of TBCs and Target Genes

Related information was manually collected for individual TBCs and their target genes to provide users a comprehensive repository about the TBC-target associations. To this end, several useful databases (e.g., UniProt, PDB) and the relevant publications that reported bioactivities of TBCs were used. Finally, detailed meta-information about each TBC-target interacting entry, such as TBC, CAS number, PubChem CID, source of compound (e.g., green, black), compound type, target gene(s) of TBC, gene symbol, gene ID, ENSEMBL ID, PDB ID, TBC bioactivity confidence and the reference were made available in the TBC2target database.

## Results

### Database Architecture

**FIGURE 2:**
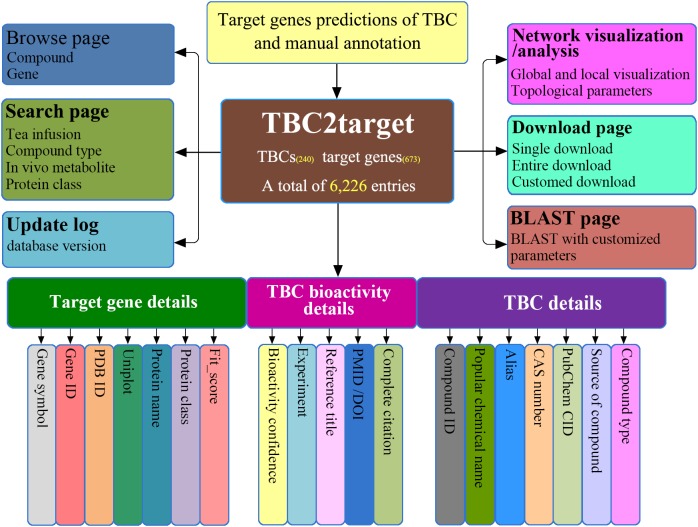
An overview of the architecture of TBC2target. The web-accessible TBC2target allows for predicted TBC-target associations to be clearly browsed, searched, downloaded and updated, under a well-organized platform framework.

TBC2target is a relational database designed on an Apache Tomcat server^3^. All the predicted TBC-target associations and the related annotation information were organized in a publicly available MySQL database^4^ as the back end, with a user friendly web-interface based on HTML, JavaScript, and CSS programming languages as the front end. As shown in the structural architecture of TBC2target (**Figure 2**), 19 data fields (e.g., CAS number, gene symbol) for each TBC-target interacting entry were presented and can be viewed in three main aspects as: (1) TBC information, (2) target gene information and (3) TBC bioactivity information, combined with several necessary database functions such as browse, search and download.

### Web Interface and Database Usage

TBC2target is developed in an easy-to-use mode, allowing for the predicted TBC-target associations to be clearly browsed, searched, downloaded, and queried using BLAST function.

#### Browse

The predicted TBC-target interacting entries can be logically viewed, as gene functional class (curated from PDB) and chemical type. These two fields can be expanded into their detailed subcategories using a PLUS clicking.

#### Search

Users can search the database using keywords, such as compound name, CAS number, PubChem CID, gene symbol, and gene ID in “compound” and “gene” fields (**Figures 1A,B**). Two logical operators (AND, OR) are deployed between these two search fields to allow targeted retrieve of specific entries. TBC2target has a fuzzy search engine that allows searching entries when a compound or a gene’s name is not clear. Upon a fuzzy search, multiple hits will be returned on the basis of spelling relevance where users can find the exact one of interest.

#### BLAST

We achieved the BLAST function in TBC2target for sequence similarity alignment. Using this function, users can upload their sequences to conduct BLAST search against TBC target genes archived in TBC2target. Upon a search, the record results will be displayed containing a list of TBC target genes with similarity to the query sequence, as well as gene accession, *e*-value, score, and useful link(s) to the corresponding gene page.

#### Download

As a publicly accessible database, TBC2target presents a page that allows the TBC-target associations to be fully downloaded as a whole or partially downloaded in a customized fashion. In addition, the whole TBC-target network file in text format is accessible for users by a link clicking.

### Data Statistics

**FIGURE 3:**
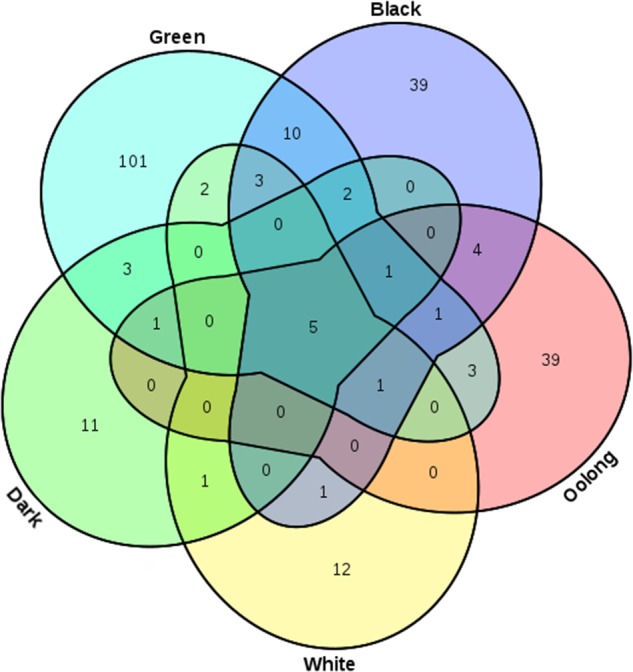
Venn diagram showing TBCs specific or common in the five main tea types (green, black, dark, oolong and white).

A total of 6,226 TBC-target interacting entries between 240 TBCs and 673 target genes were documented in TBC2target based on a computational prediction of target genes of TBCs. For the 240 TBCs (19 chemical types, see Supplementary Table S1), the bioactive confidence was manually curated from the original articles, supported by a wide range of experimental schemes such as clone 9 cells (Yen et al., 2013), rat liver homogenates (Yoshino et al., 1994) and mouse cortical neurons (Spencer et al., 2001) (see Supplementary Table S2). As indicated in **Table 1** and **Figure 3**, among the total 240 TBCs, 101, 39, 11, 39 and 12 (total 84.2%) are specific in green, black, dark, oolong and white teas, respectively, and only 5 (2.1%) are shared by all five tea types, indicating a clear chemical profile of tea related to manufacturing process and tea fermentation extent. *In vivo* metabolites of tea compounds were also included in this database. In the total 24 metabolites, several primary tea compounds, such as anthocyanin, baicali and (-)-epicatechin were prevalent for their beneficial health effects (see Supplementary Table S3). Target genes of TBCs can be classified into 99 gene functional classes. Examination of the data archived in this database revealed that several functional classes, such as oxidoreductase, hydrolase, signaling protein and transferase, were most involved in the TBC-target associations (see Supplementary Table S4), suggesting a specific functional interacting pattern associated with tea healthy mechanisms.

**Table 1:** Chemical information regarding the five main tea types.

Tea type	# of chemicals (specific^∗^)	Chemical types involved	# of reference
Green	133 (101)	19	55
Black	67 (39)	9	31
Dark	24 (11)	9	18
Oolong	55 (39)	8	17
White	25 (12)	5	17

*^∗^Number of specific TBCs in the corresponding tea type*.

**FIGURE 4:**
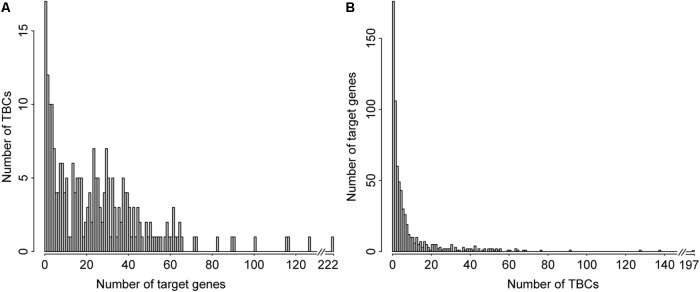
Distribution of TBC and their target genes archived in TBC2target. Histogram of the number of target genes associated with individual TBC **(A)**. Histogram of the number of TBCs associated with individual target gene **(B)**.

As shown in the histogram of the number of genes interacting with the TBCs archived in TBC2target (**Figure 4A**), 39 TBCs (16.25%) target no more than three genes and 201 (83.75%) TBCs demonstrate targeting four or more genes, with an average 26 targeted genes. The five most prevalent TBCs appeared in the database target over 100 genes; they are: prodelphinidin A-2 3′-*O*-gallate, ethyl 6-nitrocoumarin-3-carboxylyl L-theanine, oolongtheanin 3′-*O*-gallate, didesgalloyl oolonghomobisflavan B and docosahexaenoic acid, which are targeting 222, 127, 117, 116, and 101 genes, respectively. **Figure 4B** showed the histogram of the number of TBCs interacting with genes. No more than three TBC interactions were documented for 342 genes, 176 of which are targeted by only one TBC. On the contrary, 25 genes are interacting with more than 50 TBCs. It is noted that 1db1, 1pgt, 1p2s, 1p60, and 5p21 top in this list by interacting with 197, 138, 128, 92, and 78 TBCs, respectively.

### Case Study: Network Inference of Flavan-3-ols Driven Healthy Mechanisms

**FIGURE 5:**
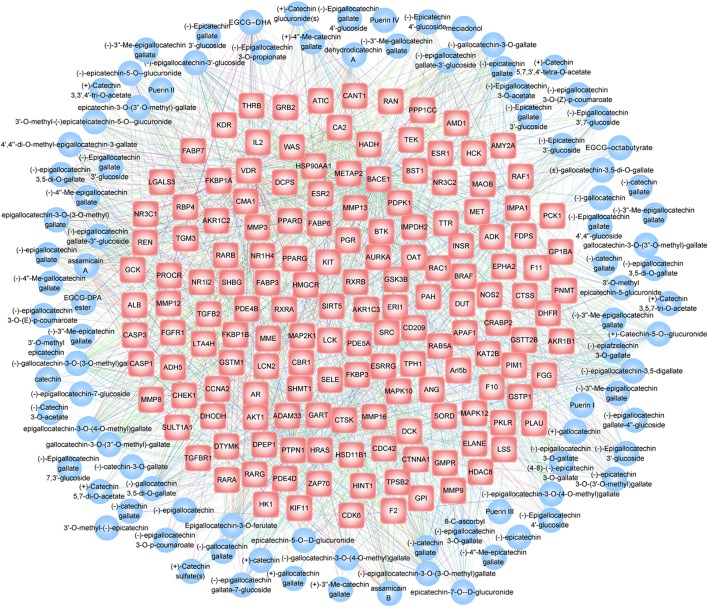
Bipartite network visualization of the TBC-target interaction relationship for the chemical type flavan-3-ols. In the network, blue circles and red hexagons correspond to TBCs and their target genes, respectively. A edge was placed between TBCs and target genes indicating TBC involvement in the regulation of the corresponding gene, with the edge color denoting gene functional class.

Flavan-3-ols, a chemical type involved in all the five tea types, contain the maximum number of chemicals (95 chemicals). Using the TBC-target associations, we reconstructed a bipartite network to describe the 1,894 interaction relationships between 95 flavan-3-ols TBCs and 156 target genes (**Figure 5**). Within the network, (-)-epigallocatechin gallate-3′′-glucoside, (-)-epigallocatechin-3-*O*-(3-*O*-methyl) gallate, (-)-epigallocatechin-3,5-digallate, epicatechin-7-*O*-β-D-glucuronide and epigallocatechin-3-*O*-(3-*O*-methyl) gallate demonstrate the highest connectivity (degree) by interacting with 88, 75, 62, 61, and 51 target genes, respectively. For target genes, the functional properties were manually annotated in this database by referring to PDB database (Burley et al., 2017). It is clearly noted that several functional classes, such as transferase (50 genes), hydrolase (37 genes), and oxidoreductase (18 genes), are prevalent by involving 77, 71, and 69 individual TBCs in the TBC-target interactions. These observations can help develop hypothesis from the global view of the interaction network by considering the knowledge of network topological parameters [e.g., “hub” node with high connectivity (Barabasi and Oltvai, 2004)]. In TBC2target, researchers can use the data in assembling this bipartite network for a specific chemical type to access novel derivations in healthy mechanisms of tea.

## Discussion

It is well known that numerous bioactive components of tea are the main sources of tea healthy function. Accumulating evidence suggests that the health-promoting benefits of tea are mediated by the critical TBC-target interactions in cellular systems (de Mejia et al., 2009). However, a complete interacting profile between TBCs and their target genes is still lacking, which limit the study of healthy mechanisms of tea. To provide a central resource for tea health research, we developed TBC2target, a database of TBC target genes based on a pharmacophore mapping approach. TBC2target not only provides a user-friendly interface to browse, search and download TBC-target association data, but it also offers several useful tools to further the use of the database.

On the basis of TBC-target associations, TBC2target can help provide network-based applications of the regulatory relationships between TBCs and their target genes. Different topological parameters of a certain TBC (or target gene) in the TBC-target network are presented in TBC2target website and have their potential usefulness. For example, a user can use the parameter “degree” to find “hub” TBCs or target genes that may play key functional role in the health-promoting system of tea (Barabasi and Oltvai, 2004). Despite of the above global network usage, the TBC-target associations of a certain chemical type (or different chemical types) can be manually extracted and network-assembled to gain novel hypothesis (see Case study as an example). From the hypothesis, a user can explore the synergistic and cross-talk effects of different TBCs. For wet experiment biologists who focus on tea health, the TBC-target network inference can provides valuable clues for their downstream experimental designs.

The TBC2target project provides an initial groundwork for distributing computationally predicted TBC-target associations in tea health research community. As described in the “Target genes prediction of TBCs” section, we used a pharmacophore mapping approach to predict target genes of TBCs. In this approach, it is clear that the number of target genes of TBCs is dependent on the Fit Score value determination. There exist several effective algorithms for target identification of small molecular compounds such as molecular docking (Shoichet et al., 2002) and 3D similarity mapping (Gong et al., 2013). Therefore, we will consider the integration of these frameworks into a single and robust pipeline to improve the data confidence. We also noted that network analysis of TBC-target interactions is useful in discovering health mechanisms of tea. However, network visualization and topological analysis presented herein are limited. In the near future, the authors will focus on a development of in-deep network analysis facilities such as TBC-target interacting motif identification and TBC–TBC synergistic pattern discovery. These strategies are promising to increase the data confidence and functional availability of this database, and promote broader interest from researchers in complementary and alternative medicine.

## Author Contributions

SZ, LZ, and YW performed the data collection and analysis, developed the database, and wrote the manuscript. JY and ML helped with the database designing and manuscript writing. SB, ZX, and C-TH provided scientific criticisms and manuscript proofreading. SZ and XW supervised the whole project and helped with the manuscript writing. All authors read and approved the final manuscript.

## Conflict of Interest Statement

The authors declare that the research was conducted in the absence of any commercial or financial relationships that could be construed as a potential conflict of interest.

## Footnotes

1 http://bbruner.org

2 https://www.certara.com

3 http://www.apache.org

4 http://www.mysql.com
