# Supplementary material for: TBC2target: A Resource of Predicted Target Genes of Tea Bioactive Compounds
Source: Front Plant Sci. 2018 Feb 22;9:211. doi: 10.3389/fpls.2018.00211 (PMC5827417; doi:10.3389/fpls.2018.00211)
Supplement: Supplementary file 1 [file Table_1.docx]

**Table S1:** A list of 19 manually curated chemical types and the related information.

| **Chemical type** | **# of Chemicals** | **Tea types involved** | **# of references** |
| --- | --- | --- | --- |
| (-)-EGCG analogs | 3 | Green | 1 |
| Amino acid | 4 | Green | 1 |
| Anthraquinones | 2 | Green, black, dark, white | 1 |
| Condensed tannins | 32 | Green, black, oolong | 7 |
| Fatty acids | 4 | Green | 2 |
| Flavan-3-ols | 95 | All five tea types | 30 |
| Flavonoids | 77 | All five tea types | 26 |
| Gingerdones | 1 | Green | 1 |
| Glucoside | 3 | Green | 1 |
| Hydrolysable tannin | 27 | Green, black, dark, oolong | 7 |
| Hydroxycinnamic acid derivatives | 1 | Green | 1 |
| Lycopene | 1 | Green | 1 |
| Phenolic acids | 43 | All five tea types | 22 |
| Short chain acids | 4 | Green, dark | 2 |
| Theaflavins | 7 | Green, black, oolong | 5 |
| Triterpene | 2 | Dark, oolong | 2 |
| Triterpenoidal saponins | 1 | Green | 1 |
| Valerolactone | 7 | Green, black, dark | 3 |
| Vitamin | 3 | Green, dark | 3 |
