# Supplementary material for: TBC2target: A Resource of Predicted Target Genes of Tea Bioactive Compounds
Source: Front Plant Sci. 2018 Feb 22;9:211. doi: 10.3389/fpls.2018.00211 (PMC5827417; doi:10.3389/fpls.2018.00211)
Supplement: Supplementary file 2 [file Table_2.docx]

**Table S2:** A list of experimental frameworks used in the original studies.

| **Experimental framework** | **# of Chemicals** | **Tea types involved** | **# of References** |
| --- | --- | --- | --- |
| (LPS)-treated macrophages and endotoxemic mice | 1 | White | 1 |
| 3-NP treated rats | 1 | Green | 1 |
| 5-week-old male ICR Mice | 2 | Green, black, oolong | 1 |
| alcohol dehydrogenase (ADH) assay | 17 | All five types | 1 |
| apc-deficient min mice/AOM-treated rats | 1 | Oolong | 1 |
| caco-2 cells | 1 | Green, black, dark | 1 |
| candida albicans | 1 | Green | 1 |
| clone 9 cells | 8 | White | 1 |
| colon cell line | 2 | Black, white | 1 |
| colonic flora | 1 | Green, black | 1 |
| DPPH assay | 44 | All five types | 9 |
| DPPH assay/tyrosinase inhibition assay | 12 | Green | 2 |
| egg-bearing adults of caenorhabditis elegans | 2 | Green | 1 |
| Escherichia coli | 10 | Black, dark, white | 3 |
| female sprague–dawley rats | 1 | Green | 1 |
| Ga1N-induced liver injuried rats | 5 | Green | 1 |
| GK rats | 2 | Black, oolong | 1 |
| H9 lymphocytes | 5 | Dark | 1 |
| HCT-116 colon cancer cells | 1 | Green, black, white | 1 |
| HeLa cells | 3 | Dark, oolong | 2 |
| HepG2 cell lines | 2 | Black, dark, white | 2 |
| HMEC cells | 4 | Dark | 1 |
| HT-29 human colon cancer cells | 6 | Green | 1 |
| human aortic endothelial cells | 1 | Green, black | 1 |
| human breast cancer cells | 1 | Black, oolong | 1 |
| Human coronaviruses | 1 | Green | 1 |
| human epidermal keratinocytes (NHEK) | 1 | Oolong | 1 |
| human erythroleukemia cells | 4 | All five types | 1 |
| human Fibrosarcoma HT1080 Cells | 2 | Green | 1 |
| human hepatoma HepG2 cells | 2 | Black | 1 |
| human histolytic lymphoma U937 | 1 | Oolong | 1 |
| human leukemia Jurkat T cells | 4 | Green | 1 |
| human liver | 1 | Green | 1 |
| human liver cells | 3 | Green, Dark | 1 |
| human SH-SY5Y cell line | 7 | Green | 1 |
| human subject | 14 | Black | 1 |
| humans | 1 | Green | 1 |
| ICR mice | 3 | Green, black, dark, oolong | 1 |
| ICR mouse atopic disease model | 1 | Oolong | 1 |
| in vitro | 40 | Green, oolong | 1 |
| in vitro digestion | 3 | Green | 1 |
| lipase assay | 3 | Dark | 1 |
| lipopolysaccharide (LPS)-stimulated murine macrophages (RAW264.7) | 2 | Green | 1 |
| LNCaP prostate cancer cells | 1 | Green | 1 |
| LPS-stimulated BV2 cells | 3 | Green, black, dark, white | 1 |
| LPS-stimulated murine macrophages | 4 | Green | 1 |
| male ICR mice | 2 | Green | 1 |
| MCF-7 breast cancer cells | 1 | Black | 1 |
| mice | 3 | Green, black | 3 |
| mouse cortical neurons | 2 | Black, white | 1 |
| mouse peritoneal macrophages | 2 | Green, black, oolong | 1 |
| mouse striatal neurons | 13 | All five types | 2 |
| murine macrophage cell line(RAW 264.7 cell line) | 4 | Black | 1 |
| pathogen-free 5-week-old female NC/Nga mice | 5 | Green, dark, oolong, white | 1 |
| platelet rich plasma (PRP) | 2 | Black, white | 1 |
| promyelocytic leukemia HL-60RG cells | 1 | All five types | 1 |
| rabbits | 2 | Green | 1 |
| rat | 2 | Black | 1 |
| rat liver homogenates | 1 | Black | 1 |
| rats | 25 | Green, black, dark, white | 8 |
| stomach cancer cell line | 1 | Green, black, white | 1 |
| the Tg2576 AD mouse model | 1 | Green | 1 |
| tumor-bearing mice | 4 | Green | 1 |
