# Supplementary material for: TBC2target: A Resource of Predicted Target Genes of Tea Bioactive Compounds
Source: Front Plant Sci. 2018 Feb 22;9:211. doi: 10.3389/fpls.2018.00211 (PMC5827417; doi:10.3389/fpls.2018.00211)
Supplement: Supplementary file 3 [file Table_3.docx]

**Table S3:** A list of 10 in vivo metabolites and the related information.

| **In vivo metabolites** | **# of Chemicals** | **Tea types involved** | **# of references** |
| --- | --- | --- | --- |
| In vivo metabolites of Rutin | 1 | Green, black | 1 |
| In vivo metabolites of Quercetin | 4 | Black | 1 |
| In vivo metabolites of Poncirin | 1 | Black | 1 |
| In vivo metabolites of Myricetin | 1 | Black | 7 |
| In vivo metabolites of Catechin | 1 | White | 2 |
| In vivo metabolites of Baicali | 1 | Green, white | 37 |
| In vivo metabolites of Anthocyanin | 1 | Green, black, dark | 39 |
| In vivo metabolites of (+)-Catechin | 11 | Green, black, | 1 |
| In vivo metabolites of (-)-epicateicatechin | 1 | Green | 1 |
| In vivo metabolites of (-)-epicatechin | 2 | Green, black | 12 |
