# Supplementary material for: TBC2target: A Resource of Predicted Target Genes of Tea Bioactive Compounds
Source: Front Plant Sci. 2018 Feb 22;9:211. doi: 10.3389/fpls.2018.00211 (PMC5827417; doi:10.3389/fpls.2018.00211)
Supplement: Supplementary file 4 [file Table_4.docx]

**Table S4:** A complete list of 91protein class and the related information.

| **Protein class** | **# of genes** | **# of interacting chemicals** | **# of chemical types** |
| --- | --- | --- | --- |
| AMINOPEPTIDASE | 2 | 44 | 12 |
| \| ANDROGEN RECEPTOR \| \| --- \| | 1 | 44 | 10 |
| ANGIOGENIN | 1 | 1 | 1 |
| ANTIMICROBIAL PROTEIN | 1 | 3 | 3 |
| APOPTOSIS | 2 | 67 | 11 |
| APOPTOSIS HYDROLASE | 1 | 44 | 8 |
| ASPARTIC PROTEINASE | 1 | 5 | 4 |
| ATTRACTANT | 1 | 1 | 1 |
| [BILIVERDIN-IX BETA REDUCTASE](http://www.rcsb.org/pdb/search/smartSubquery.do?smartSearchSubtype=StructureKeywordsQuery&display=true&struct_keywords.pdbx_keywords.value=BILIVERDIN-IX%20BETA%20REDUCTASE&struct_keywords.pdbx_keywords.comparator=contains) | 1 | 1 | 1 |
| BLOOD CLOTTING | 3 | 15 | 8 |
| BLOOD CLOTTING/HYDROLASE INHIBITOR | 1 | 8 | 4 |
| CALCIUM-BINDING PROTEIN | 1 | 3 | 3 |
| CELL CYCLE | 2 | 69 | 11 |
| CELL CYCLE COMPLEX | 1 | 12 | 5 |
| CELL CYCLE/TRANSFERASE | 1 | 40 | 2 |
| CHAPERONE | 4 | 82 | 12 |
| CHAPERONE PROTEIN | 1 | 8 | 4 |
| CIS-TRANS ISOMERASE | 1 | 30 | 10 |
| COMPLEX (GTP-BINDING/GTPASE ACTIVATION) | 1 | 10 | 5 |
| COMPLEX (HYDROLASE/INHIBITOR) | 1 | 7 | 3 |
| COMPLEX (METALLOPROTEASE/INHIBITOR) | 1 | 4 | 2 |
| COMPLEX (PHOSPHOTRANSFERASE/RECEPTOR) | 1 | 1 | 1 |
| COMPLEX (PROTEIN/PROTEIN) | 1 | 35 | 12 |
| COMPLEX (SERINE PROTEASE/INHIBITOR) | 1 | 34 | 7 |
| COMPLEX (TRANSFERASE/CYCLIN2) | 1 | 6 | 5 |
| COMPLEX (TRANSFERASE/SULFASALAZINE) | 1 | 2 | 2 |
| COMPLEX HYDROLASE/INHIBITOR | 1 | 1 | 1 |
| COMPLEX(SERINE) | 1 | 8 | 3 |
| CYTOKINE | 2 | 15 | 8 |
| CYTOSKELETON | 1 | 1 | 1 |
| DNA BINDING PROTEIN | 3 | 4 | 4 |
| ELECTRON TRANSPORT | 1 | 1 | 1 |
| FLAVOPROTEIN | 1 | 1 | 1 |
| GENE REGULATION | 3 | 87 | 14 |
| GROWTH FACTOR | 1 | 6 | 4 |
| GTP-BINDING PROTEIN | 1 | 1 | 1 |
| HORMONE GROWTH FACTOR RECEPTOR | 6 | 32 | 13 |
| HORMONE HORMONE ACTIVATOR | 1 | 1 | 1 |
| HORMONE RECEPTOR | 3 | 22 | 10 |
| HUMAN PROGESTERONE RECEPTOR | 1 | 7 | 3 |
| HYDROLASE | 53 | 192 | 15 |
| HYDROLASE ACID PROTEINASE | 1 | 4 | 3 |
| HYDROLASE HYDROLASE INHIBITOR | 1 | 2 | 2 |
| HYDROLASE SERINE PROTEASE | 1 | 6 | 3 |
| HYDROLASE SERINE PROTEINASE | 2 | 40 | 8 |
| IMMUNE SYSTEM | 3 | 11 | 7 |
| IMMUNE SYSTEM MEMBRANE PROTEIN | 1 | 11 | 6 |
| INHIBITOR / APOPTOSIS | 1 | 1 | 1 |
| ISOMERASE | 6 | 80 | 12 |
| ISOMERASE / TRANSFERASE | 1 | 1 | 1 |
| KINASE | 2 | 11 | 7 |
| LACTOYLGLUTATHIONE LYASE | 1 | 1 | 1 |
| LIGASE | 3 | 7 | 4 |
| LIPID BINDING PROTEIN | 6 | 34 | 10 |
| LYASE | 9 | 78 | 11 |
| MEMBRANE PROTEIN | 2 | 2 | 2 |
| METAL BINDING PROTEIN | 1 | 1 | 1 |
| METALLOPROTEASE | 1 | 1 | 1 |
| METALLOPROTEINASE | 1 | 18 | 10 |
| NUCLEAR PROTEIN | 1 | 3 | 2 |
| NUCLEAR RECEPTOR | 3 | 35 | 11 |
| NUCLEAR TRANSPORT PROTEIN COMPLEX | 1 | 1 | 1 |
| ONCOGENE PROTEIN | 2 | 201 | 16 |
| OXIDOREDUCTASE | 30 | 166 | 16 |
| OXIDOREDUCTASE (CH-NH(D)-NAD OR | 1 | 6 | 6 |
| PEPTIDE BINDING PROTEIN | 1 | 6 | 6 |
| PHOSPHOTRANSFERASE | 3 | 49 | 10 |
| PLASMA PROTEIN | 1 | 18 | 10 |
| PROTEIN BINDING | 1 | 2 | 2 |
| PROTEIN KINASE INHIBITOR | 1 | 59 | 6 |
| PROTEIN TRANSPORT | 3 | 37 | 5 |
| RECEPTOR | 1 | 1 | 1 |
| RETINOIC-ACID TRANSPORT | 1 | 1 | 1 |
| RETINOL TRANSPORT | 1 | 18 | 8 |
| RNA BINDING PROTEIN | 1 | 1 | 1 |
| SERINE PROTEASE | 1 | 5 | 3 |
| SERUM PROTEIN | 1 | 13 | 5 |
| SIGNALING PROTEIN | 7 | 139 | 14 |
| SUGAR BINDING PROTEIN | 2 | 21 | 4 |
| SULFOTRANSFERASE | 1 | 1 | 1 |
| TRANSCRIPTION | 13 | 48 | 12 |
| TRANSCRIPTION RECEPTOR/COACTIVATOR | 1 | 7 | 3 |
| TRANSCRIPTION REGULATION | 1 | 6 | 4 |
| TRANSCRIPTION REGULATION RECEPTOR | 1 | 1 | 1 |
| TRANSCRIPTION REGULATOR | 1 | 7 | 5 |
| TRANSFERASE | 78 | 209 | 19 |
| TRANSFERASE / TRANSFERASE INHIBITOR | 1 | 1 | 1 |
| TRANSFERASE ACTIVATOR | 1 | 8 | 4 |
| TRANSFERASE BLOOD CLOTTING | 1 | 2 | 1 |
| TRANSFERASE CELL CYCLE | 1 | 4 | 3 |
| TRANSFERASE GLUTATHIONE | 1 | 37 | 7 |
| TRANSFERASE SIGNALING PROTEIN | 1 | 25 | 7 |
| TRANSFERASE BLOOD CLOTTING | 1 | 52 | 8 |
| TRANSPORT PROTEIN | 4 | 58 | 7 |
| TRANSPORT THYROXINE | 1 | 4 | 2 |
| TYROSINE KINASE | 2 | 27 | 6 |
